# Melatonin in Apples and Juice: Inhibition of Browning and Microorganism Growth in Apple Juice

**DOI:** 10.3390/molecules23030521

**Published:** 2018-02-27

**Authors:** Haixia Zhang, Xuan Liu, Ting Chen, Yazhen Ji, Kun Shi, Lin Wang, Xiaodong Zheng, Jin Kong

**Affiliations:** College of Horticulture, China Agricultural University, Beijing 100193, China; zhx2323a@163.com (H.Z.), xuan0414@sina.com (X.L.), 18811702979@163.com (T.C.), zyp001@163.com (Y.J.), 15600912620@163.com (K.S.), wl1808@sina.com (L.W.), zheng.xiao.d@163.com (X.Z.)

**Keywords:** melatonin, apple, juice processing, antioxidant, anti-microorganism

## Abstract

Synthetic melatonin (*N*-acetyl-5-methoxytryptamine, MT) is popular in the US and Asian markets as a health supplement. Here, we identified a naturally occurring melatonin source in apple juice. Melatonin was present in all 18 apple cultivars tested. The highest melatonin level of the edible part of apple was detected in the apple peel. The melatonin content in ‘Fuji’ apple juice is comparable to the level of its flesh. Melatonin was consumed during the process of juicing due to its interaction with the oxidants. Melatonin addition significantly reduced the juice color change to brown (browning). The mechanism is that melatonin scavenges the free radicals, which was indicated by the ASBT analysis; therefore, inhibiting the conversion of *o*-diphenolic compounds into quinones. Most importantly, melatonin exhibited powerful anti-microorganism activity in juice. The exact mechanisms of this action are currently unknown. These effects of melatonin can preserve the quality and prolong the shelf life of apple juice. The results provide valuable information regarding commerciall apple juice processing and storage.

## 1. Introduction

Apple is one of the top four favorite fruits in the world. A total of 63,407,407 metric tons of apple worth approximately $23.5 billion are consumed yearly [1,2,3]. Apple is very popular as a healthy fruit and it contains multiple minerals, vitamins, and phytochemicals, including carotenoids, flavonoids, polyphenolic compounds, and phenolic acids [4,5,6,7,8,9,10]. Apple juices are also important dietary sources of these bioactive compounds [10,11]. It was reported that consuming apples and apple juice effectively reduces the risk of several cancers, cardiovascular disease, and asthma [12]. However, the underlying mechanisms remain elusive. Since melatonin is a potent antioxidant, in the current study, the antioxidant capacity of melatonin in apple and apple juice was systemically investigated. 

Melatonin was first identified in plants in 1995; thereafter, it was found that melatonin existed in variety of plants and plant products [13,14,15,16,17,18]. In 2013, we firstly reported that melatonin was also present in apple fruit [19]. As a potent reactive oxygen species (ROS) and antioxidant, melatonin may be a major contributor regarding the health effects of apple, including its antioxidant, anticancer, antitumor, anti-inflammatory, anti-aging, anti-diabetic, antiviral and neuroprotective activities [20]. It also has therapeutic potential for respiratory, colonic, autoimmune, adult-onset chronic non-communicable diseases, ischemic brain and cardiovascular injury, circadian disturbance, stroke, steatohepatitis, etc. [21,22,23,24,25,26,27,28,29,30,31,32,33,34,35,36,37,38,39,40,41]. Melatonin can efficiently scavenge a variety of ROS including hydrogen peroxide (H_2_O_2_), hydroxyl radical (HO•), nitric oxide (NO•), singlet oxygen (^1^O_2_) and superoxide anion (O^2–^•) [42]. Compared with other classical antioxidants such as vitamin C, vitamin E and glutathione, melatonin is amphiphilic and can freely diffuse into different organelles. In addition, its metabolites, such as *N*^1^-acetyl-*N*^2^-formyl-5-methoxykynuramine (AFMK), *N*-acetyl-5-methoxykynuramine (AMK) and 6-hydroxymelatonin also possess the antioxidant activities, which amplify the capacity of melatonin as an antioxidant. The collective effects of melatonin and its metabolites are referred as a cascade reaction of melatonin [42,43,44,45,46,47].

Compared to synthetic melatonin, phyto-melatonin [48] may be more welcome by consumers. One of the natural melatonin sources are the fruits and their juices. Compared to the popularity of apple and its juice there is little information regarding the melatonin content in apple fruit of different cultivars and their juice. Especially, in ‘Fuji’ fruits, melatonin may be lost during the processing procedure or be consumed by the exposure to oxidants in air. No report is available on the natural melatonin level left after apple juicing. Therefore, it is difficult to know whether exogenous melatonin needs to be added to preserve the quality of the juice. 

During the juicing process, *o*-diphenolic compounds are converted to quinones in the damaged cells and this results in the color of juice changing to brown. Inhibition of this oxidative reaction reduces the juice browning. Numerous studies on reducing color changes in apple juice have been reported [49,50,51,52,53,54]. Inhibitors of apple juice browning are divided into six groups: reducing molecules, chelating agents, complexing substances, acidulants, enzyme inhibitors, and enzyme treatments [49,55,56]. However, very few of these inhibitors are practically used in the food industry due to concerns about off-flavour and odours, food safety, and economic feasibility [57]. Therefore there is an increasing interest in identifying additional inhibitors which are safe, cheap, and abundant to enhance the stability of juice which is susceptible to browning. 

As a powerful antioxidant, melatonin may be a first choice for healthy food supplements. In the current study, ‘Fuji’ apple was selected for apple juicing, since in China 70% of the apple production is ‘Fuji’ [58]. Melatonin levels in the apple peel and flesh of 18 cultivars were tested and also exogenous melatonin was supplemented to ‘Fuji’ apple juice during its processing to explore its ability to reduce the browning and inhibit microbial growth. Its antioxidant capacity was also checked. Our research should contribute to the application of melatonin as an anti-browning molecule and health care component in the apple juice industry. 

## 2. Results

### 2.1. The Content of Melatonin and Total Phenols in ‘Fuji’ and ’Granny Smith’ Apple Fruit

**Figure 1:**
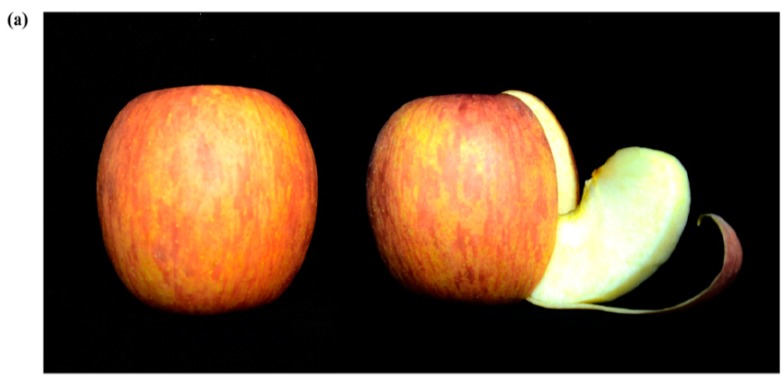

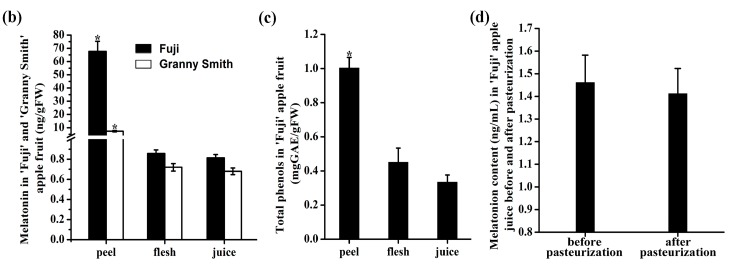
Melatonin and total phenol content in apple. (**a**) The ‘Fuji’ apple peel and flesh samples. (**b**) Melatonin content in peel, flesh, and juice of the ‘Fuji’ and ’Granny Smith’ apple. (**c**) The total phenol content in the peel, flesh, and juice of ‘Fuji’ apple. (**d**) The effect of pasteurization on the content of melatonin in ‘Fuji’ apple juice. Data are means ± SD of three independent studies, and three duplicates for each analysis. * indicates significant difference among treatments based on LSD multiple comparison (*p* < 0.05).

All the juice melatonin comes from the flesh since the melatonin from the peel may not be incorporated into the juice. The results showed that the highest melatonin level was in the peel of ‘Fuji’ apple (67.627 ng/gFW) and ’Granny Smith’ apple (7.37 ng/gFW), which was nearly 79- and 10- fold higher than the levels in their flesh (0.857 ng/gFW and 0.719 ng/gFW) and 83-times and 11-fold higher than that in their juices (0.814 ng/gFW and 0.680 ng/gFW), respectively (Figure 1b).

The content of total phenols in the ‘Fuji’ apple peel (1.00 mgGAE/gFW) was nearly 2-times and 3-fold higher than that in their flesh (0.45 mgGAE/gFW) and fruit juice (0.33 mgGAE/gFW), respectively (Figure 1c). Obviously, both the melatonin and total phenol in apple peel were significantly higher than that in their flesh and juice, but there was no significant difference in the melatonin and total phenol content between the flesh and fruit juice.

### 2.2. The Effect of Pasteurization on the Melatonin Content in Apple Juice

Pasteurization seems not influence melatonin levels in apple juice. Its levels were 1.46 and 1.41 ng/mL before and after pasteurization, respectively. There was no significant difference (*p* < 0.05) (Figure 1d). 

### 2.3. The Melatonin Content in Different Apple Cultivars

**Figure 2:**
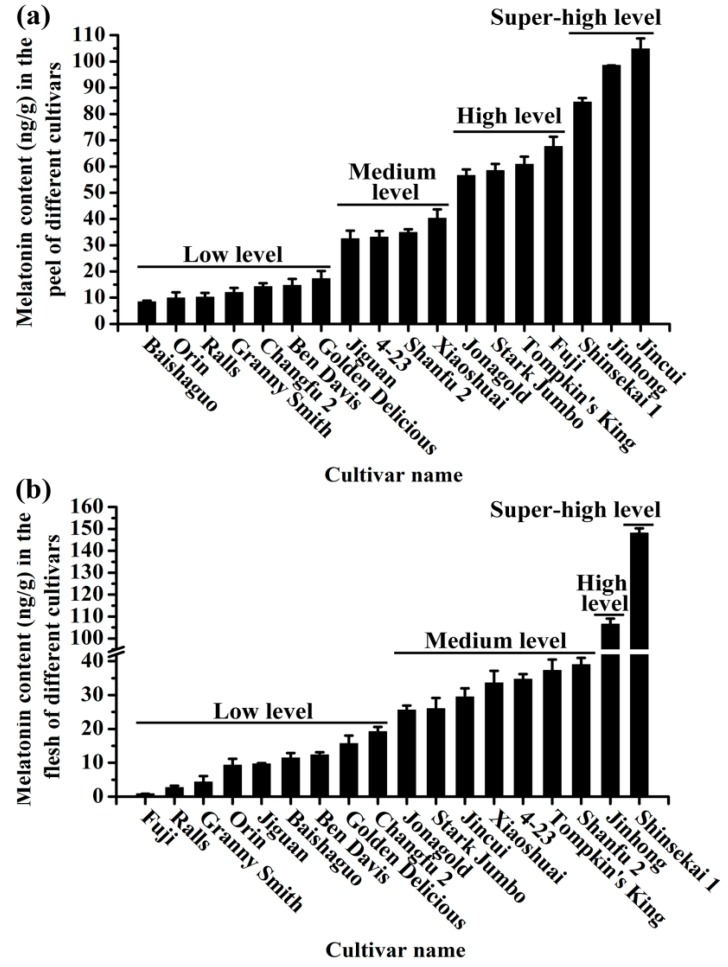
The melatonin levels in the peel and flesh of different apple cultivars. (**a**) The melatonin content in the peel of different cultivars. (**b**) The melatonin content in the flesh of different cultivars. Data are means ± SD of three independent studies, and three duplicates for each analysis.

The melatonin contents in apple peel and flesh among the 18 commercial cultivars varied from 0.86 ng/g to 148.11 ng/g (Figure 2). For most of the cultivars, there was a good correlation between the melatonin in the peel and the flesh. If there was a high level in the peel, generally speaking, there was a high level in the flesh.

Based on the melatonin levels in apple peel, these cultivars were divided into four groups including super-high, high, medium and low level groups (Table 1 in the Supplementary Data). The cultivars ‘Baishaguo’, ‘Orin’, ‘Ralls’, ‘Granny Smith’, ‘Changfu 2’, ‘Ben Davis’ and ‘Golden Delicious’ with melatonin ranging from 8.37 to 17.18 ng/g were classified as the low melatonin group; ‘Jiguan’, ‘Shanfu 2’, ‘4-23’ and ‘Xiaoshuai’ with melatonin levels from 32.43 to 40.3 ng/g were the medium level group; ‘Jonagold’, ‘Stark Jumbo’, ‘Tompkin’s King’ and ‘Fuji’ with melatonin from 56.55 to 67.63 ng/g were the high level group and in the super-high level group, the melatonin levels were from 87.28 to 105.97 ng/g. For example, the melatonin level in the peel of ‘Shinsekai 1’ was 87.28 ng/g, in ‘Jinhong’ it was 102.64 ng/g and in ‘Jincui’ it was 105.97 ng/g, which were 1.25-, 1.46- and 1.55-fold higher than that in the most popular ‘Fuji’ apple (67.63 ng/g), respectively (Figure 2a).

Based on the apple flesh melatonin levels, these cultivars were also classified into four groups (Table 2 in the Supplementary Data) like the peel. In the low level group, the melatonin content in the flesh of nine cultivars (‘Fuji’, ‘Ralls’, ‘Granny Smith’, ‘Orin’, ‘Jiguan’, ‘Baishaguo’, ‘Ben Davis’, ‘Golden Delicious’ and ‘Changfu 2’) ranged from 0.86 ng/g to 19.22 ng/g; in the medium level group, which included seven apple cultivars (‘Jonagold’, ‘Stark Jumbo’, ‘Jincui’, ‘Xiaoshuai’, ‘4-23’, ‘Tompkin’s King’ and ‘Shanfu 2’), melatonin ranged from 25.55 ng/g to 39.0 ng/g; ‘Jinhong’ and ‘Shinsekai 1’ were both the only cultivar in the high and super-high level groups, with 106.54 ng/g and 148.11 ng/g melatonin, respectively (Figure 2b). 

### 2.4. The Melatonin Content Reduced during Juicing Procedure and Storage Time

**Figure 3:**
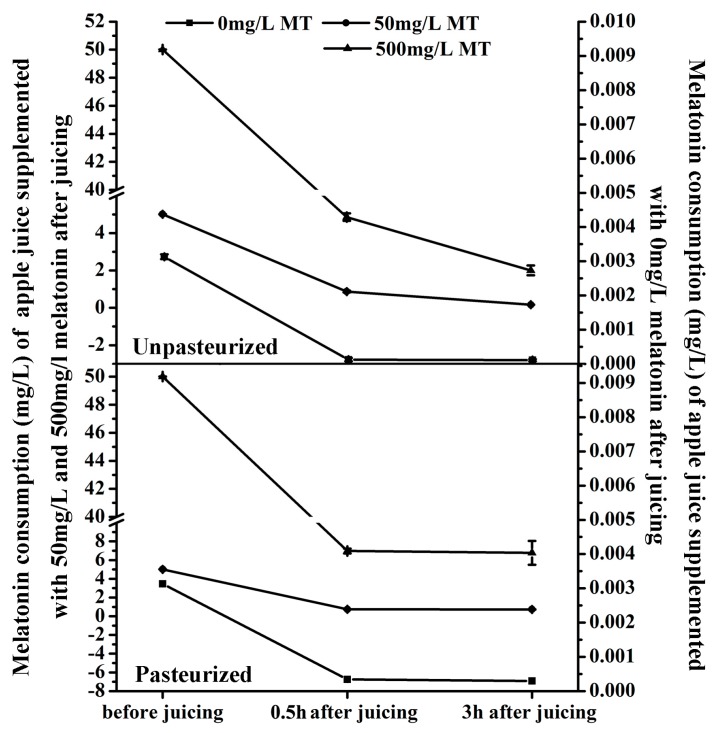
Effects of pasteurization on melatonin levels in apple juice. Melatonin content of unpasteurized and pasteurized ‘Fuji’ apple juice supplemented with 50 or and 500 mg/L MT (melatonin) respectively. Data are means ± SD of three independent studies, and three duplicates for each analysis.

The melatonin content in ‘Fuji’ apple was detected at three time points: just after juicing, just after pasteurization (only for the pasteurized juice, pasteurization was carried out just after juicing) and three hours after juicing (for both the pasteurized juice and unpasteurized juice). The melatonin level was reduced sharply during the juicing process and then declined gradually during storage, whether the juice was pasteurized or not (Figure 3). 

For ‘Fuji’ apple, its juice yield (the ratio of final apple juice volume and the weight of apple fruit) is around 70%. The melatonin solution was added into the juice in 7:100 (*v*/*w*) before juicing. It means the supplemented melatonin in the apple juice was diluted nearly 10 times. Even without any consumption or loss of the supplemented melatonin, the supplemented melatonin is only 5 mg/L or 50 mg/L left after juicing, respectively. 

The average weight of ‘Fuji’ fruit was about 150 g including 3.0 g peel with an average melatonin content of 67.63 ng/g (total melatonin was around 202.89 ng) and 147.0 g flesh with an average melatonin level of 0.857 ng/g (total melatonin was about 125.98 ng) (Figure 1b). Therefore the average total melatonin content of one ‘Fuji’ apple (including peel and flesh) was about 329 ng. The juice yield of an apple fruit was 105 mL and the average melatonin content of apple juice before juicing was about 3.13 ng/mL (0.00313 mg/L). 

In this experiment, the average water for each apple fruit was 10 mL, which was used as blank control for the melatonin supplementation. As shown in Figure 3, 0.5 h after juicing, the melatonin content in the unpasteurized juice of the control decreased from 3.13 ng/mL to 0.125 ng/mL, a reduction of 96% (Figure 3a). When 50 mg/L and 500 mg/L melatonin were supplemented before juicing, the melatonin levels also reduced from 5 mg/L and 50 mg/L to 0.870 mg/L (reduction by about 83%) and 4.858 mg/L (reduction by about 90%), respectively. In the pasteurized juice, the decrease in melatonin content was similar to the control (Figure 3b). It decreased from 3.13 ng/mL to 0.344 ng/mL (reduction by about 89%) in the control, and from 5 mg/L and 50 mg/L to 0.739 mg/L (reduction by about 85%) and 6.971 mg/L (reduction by about 86%) in 50 mg/L and 500 mg/L melatonin supplemented juice, respectively. Obviously, juicing resulted in a significant melatonin decrease in apple juice, but pasteurization didn’t make a significant difference compared with the unpasteurized apple juice.

After 3 hours’ storage, in the unpasteurized juice the melatonin levels were reduced from 0.125 ng/mL, 0.870 mg/L and 4.858 mg/L to 0.109 ng/mL, 0.166 mg/L and 2.002 mg/L (reductions by about 13%, 80% and 59%) in the blank control and groups treated with 50 mg/L and 500 mg/L melatonin supplementation, respectively (Figure 3a). However, in the pasteurized juice, melatonin levels were only reduced from 0.344 ng/mL, 0.739 mg/L and 6.971 mg/L to 0.296 ng/mL, 0.726 mg/L and 6.780 mg/L (reductions by about 14%, 2%, and 3%) in control and the two groups supplemented with melatonin, respectively (Figure 3b).

### 2.5. The Effect of Exogenous Melatonin on the Browning for ‘Fuji’ Apple Juicing 

**Figure 4:**
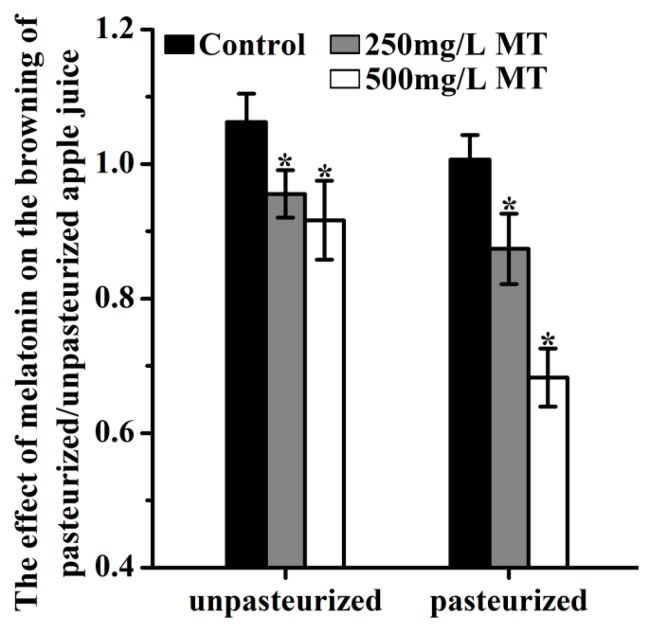
The effect of melatonin on the browning of unpasteurized or pasteurized ‘Fuji’ apple juice. MT was for melatonin. Data are means ± SD of three independent studies, and three duplicates for each sample. * indicates significant differences among treatments, based on LSD multiple comparison (*p* < 0.05).

Addition of 250 mg/L and 500 mg/L melatonin before juicing significantly reduced the browning. This was indicated by the changes of browning index and observed whether the juice was pasteurized or not (Figure 4). 

**Figure 5:**
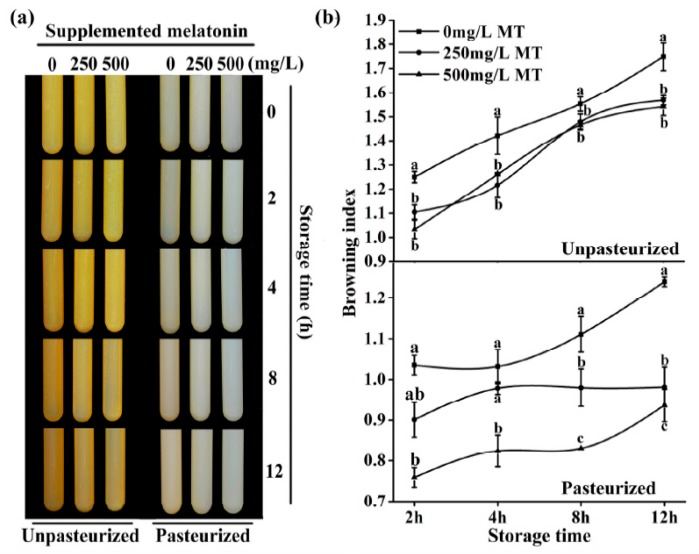
The effect of melatonin on the browning of ‘Fuji’ apple juice at different storage time. (**a**) Photo of the ‘Fuji’ apple juice supplemented with 250 or 500 mg/L MT (melatonin) during storage with or without pasteurization. (**b**) The browning index of the ‘Fuji’ apple juice supplemented with 250 mg/L or 500 mg/L MT (melatonin) during storage with or without pasteurization. Data are means ± SD of three independent studies, and three duplicates for each analysis. Lower-case letters indicate significant differences among treatments based on LSD multiple comparison (*p* < 0.05).

The browning index for controls was 1.062 and 1.007 in unpasteurized and pasteurized juices, respectively. In the 250 mg/L added melatonin group, they were 0.956 and 0.874 in the unpasteurized and pasteurized juices respectively. In the 500 mg/L added melatonin group, the indexes were 0.917 and 0.683, respectively, in unpasteurized and pasteurized juices. After 12 hours of storage, the browning was further increased in all groups. Obviously, the browning of unpasteurized juice was more severe than that of pasteurized juice. Both doses of melatonin treatment significantly decreased the juice browning at this time point too (Figure 5)

### 2.7. The Antioxidant Capacity of Exogenous Melatonin in Unpasteurized and Pasteurized ‘Fuji’ Apple Juice during Storage 

**Figure 6:**
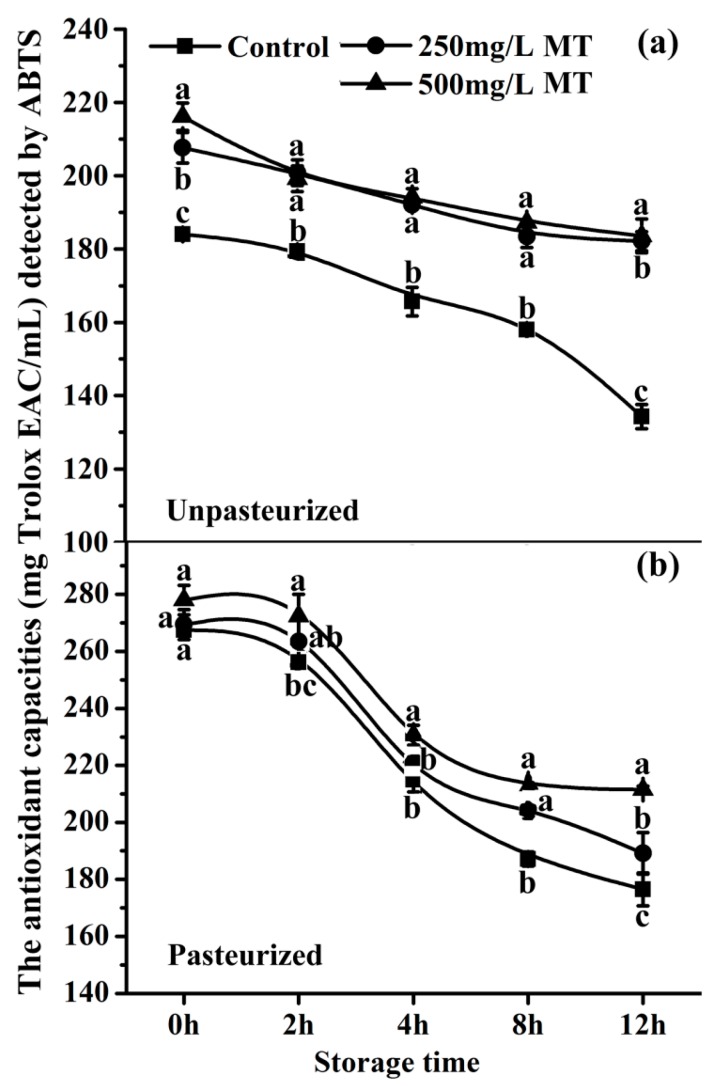
The effect of melatonin of 250 or 500 mg/L on the antioxidant capacities of ‘Fuji’ apple juice. (**a**) and (**b**) The antioxidant capacities of ‘Fuji’ apple juice with 250 or 500 mg/L MT (melatonin) with or without pasteurization compared to the control respectively. Antioxidant capacity was evaluated by ABTS analysis. Data are means ± SD of three independent studies, and three duplicates for each analysis. Lower-case letters indicate significant differences among treatments based on LSD multiple comparison (*p* < 0.05).

The ABTS method was applied to detect the antioxidant capacities in unpasteurized and pasteurized ‘Fuji’ apple juice with or without 250 mg/L and 500 mg/L melatonin treatment. During storage, the antioxidant capacity of the unpasteurized control juice gradually decreased during the first 8 hours. Thereafter, this decrease was accelerated from 8 (162.09 mg Trolox EAC/mL) to 12 h (134.29 mg Trolox EAC/mL) (Figure 6a). A different pattern was observed in pasteurized control and 250 and 500 mg/L melatonin treatment juices. The antioxidant capacities of these juices had a sharp decrease from 2 (266.07, 275.59 and 284.52 mg Trolox EAC/mL respectively) to 4 h (208.06, 210.25 and 222.95 mg Trolox EAC/mL respectively), after that, the changes were very slow (Figure 6b). Melatonin supplementation at 500 mg/L significantly increased the antioxidant capacities in both pasteurized and unpasteurized juices, compared to the control or 250 mg/L melatonin groups (Figure 6). 

### 2.8. The Effects of Exogenous Melatonin on the Microorganism Growth in ‘Fuji’ Apple Juice during Storage

**Figure 7:**
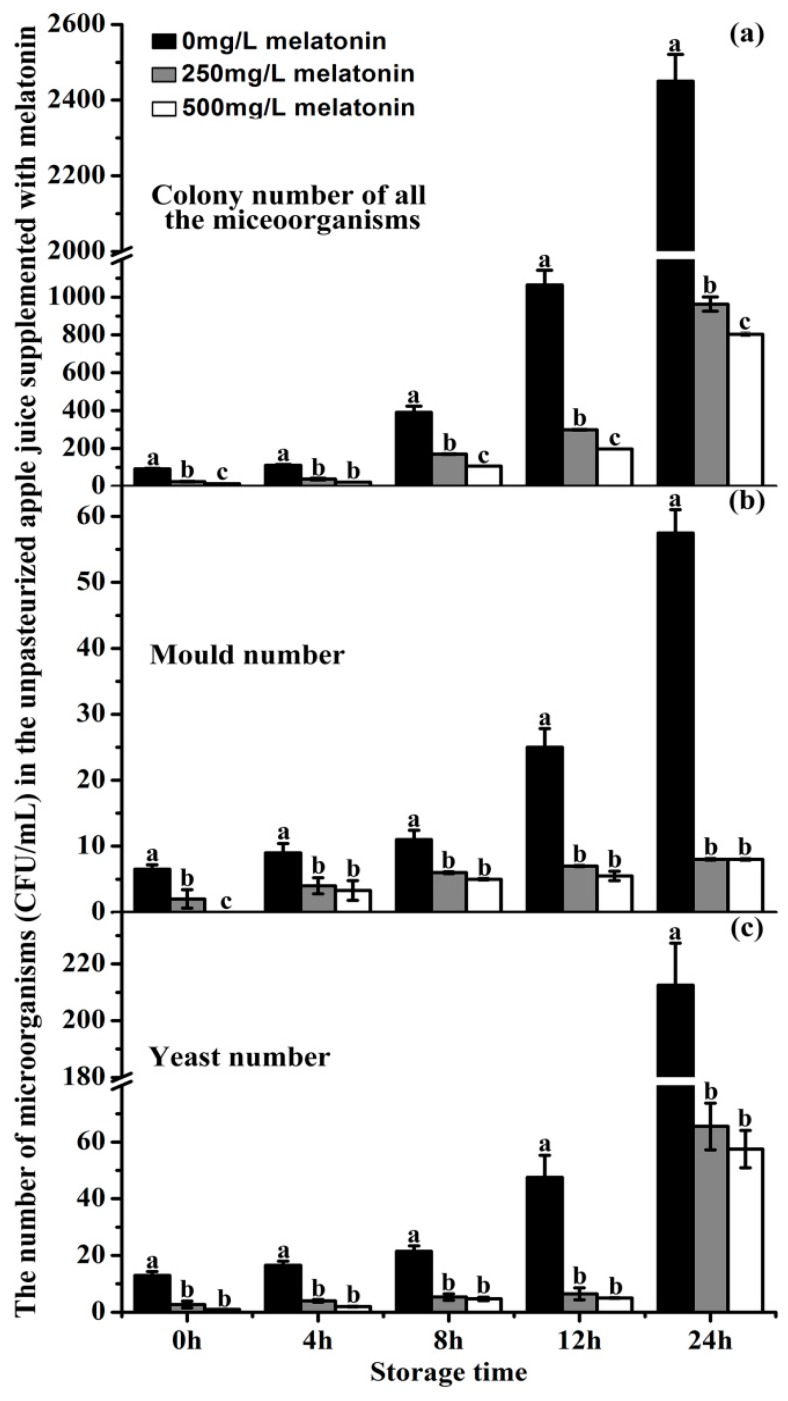
Effects of melatonin on microorganism growth in the unpasteurized ‘Fuji’ apple juice (**a**), (**b**) and (**c**) The number of total colonies, moulds and yeasts in the unpasteurized ‘Fuji’ apple juice with 250 or 500 mg/L MT (melatonin), respectively. Data are means ± SD of three independent studies, and three duplicates for each analysis. Lower-case letters indicate significant differences among treatments based on LSD multiple comparison (*p* < 0.05).

The capacity of melatonin to inhibit microorganism growth was tested in unpasteurized ‘Fuji’ apple juice by counting the colony numbers of yeast, moulds and other microorganisms. As expected, the microorganisms proliferated rapidly during the 24 h of juice storage. In the control juice, at 12 h storage, the number of microorganisms had already exceeded its quality guarantee limits. In details, its total colony number of microorganisms at 12 h storage were 1064 CFU/mL which was roughly 12-fold higher than that at 0 h (91 CFU/mL) (Figure 7a). Both 250 and the 500 mg/L melatonin treatments significantly inhibited microorganism growth (including yeasts, moulds and bacteria), in juice. The total microorganisms in 250 and 500 mg/L melatonin-treated juices were 299 CFU/mL and 197 CFU/mL, respectively, which were only 28 and 19% of the control regarding the number of microorganisms.

At 12 h storage, the mould colony number and yeast number in the control juice were roughly 4-fold higher than those at time 0 (Figure 7b,c). In contrast, in both added melatonin groups, the mould colony numbers were reduced to 28% (7 CFU/mL) and 22% (5.5 CFU/mL) compared to the control (25 CFU/mL), respectively (Figure 7b); the yeast numbers were decreased to 14% (6.5 CFU/mL) and 11% (5 CFU/mL) related to the control (47.5 CFU/mL), respectively (Figure 7c). The inhibition of exogenous melatonin on microorganism growth was detected at all time points analyzed. It appeared that the inhibitory effect of 500 mg/L melatonin on microorganism growth was more potent than that of 250 mg/L (Figure 7). 

## 3. Discussion

Melatonin is a popular food supplement with human health benefits [59]. It was first reported to be present in apple fruit in 2013 [19]; therefore, it is expected that apple juice should be rich in melatonin. If so, apple juice could serve as an alternative source of naturally occurring melatonin which may be more welcome by the consumers compared to the synthetic version. In the present study, the melatonin content in apple peel and flesh were tested in variety of cultivars (18 in total) (Figure 2). The apple cultivars rich in melatonin apple could be selected to produce ‘apple juice rich in melatonin’ in the future. Melatonin content in these cultivars exhibited substantial variations. Two cultivars (‘Jinhong’ and ‘Shinsekai 1’) had the highest melatonin content among others (Figure 2). These two apple cultivars will be candidates for producing ‘melatonin-rich juice’ in the future. The content of melatonin as well as the total phenols in ‘Fuji’ apple peel was significantly higher than that in apple flesh and juice (Figure 1b,c). It is well known that apple peel contains higher levels of antioxidant chemicals such as phenols and flavonoids than flesh and therefore has higher antioxidant activity [60,61,62,63]. During the coloration in the final stage of apple development, the high intensity of light during the daytime and the low temperature at night probably contribute to the accumulation of these antioxidants. Melatonin levels in the peel are likely induced by environmental stressors. This is supported by the previous reports that melatonin content increased when fruits are subject to stressors like cold or high light intensity [64,65,66,67,68,69,70,71]. 

Compared to the melatonin level in peel (67.63 ng/gFW), the melatonin content in Fuji apple juice (0.814 ng/gFW) is comparable to that in flesh (0.857 ng/gFW), (Figure 1b). A similar result was also observed in ‘Granny Smith’ apple juice. It appears that the juice melatonin level was decided by the flesh melatonin. However, the possibility of melatonin losses during the processing of juice could not be ignored. Initially, we thought that only the free melatonin was left in juice and the bound melatonin was lost. In addition, incomplete cell breakage might also result in melatonin being lost. Our results indicated that melatonin addition (250 mg/L and 500 mg/L) before the juicing process also resulted in 80~90% melatonin loss. It is obvious that this melatonin loss during juice processing is due to the reactions of melatonin with oxidants. This was further proved by the protective effects of melatonin on juice browning. When the juice was exposed to the air it rapidly changed color to brown (referred to as browning). The majority of clinical trials on the therapeutic usefulness of melatonin in different fields of medicine have shown very low toxicity of melatonin over a wide range of doses [72], and melatonin is also recommended to be used as a health food supplement in China [73]. In addition, the commercial melatonin tablets contain 1 mg, 3 mg or 5 mg melatonin. Therefore, addition of synthetic melatonin as a healthy supplement to apple juice would be legal.

This browning process is probably via the conversion to quinones of *o*-diphenolic compounds which is the main oxidative reaction occurring during fruit color change [74] Melatonin addition at the concentrations of 250 and 500 mg/L significantly reduced the browning in unpasteurized apple juice, during juicing and storage, respectively (Figure 4 and Figure 5). This was due to the potent antioxidant capacity of melatonin indicated by the free radical scavenging ABTS analysis (Figure 6a). Likewise, for the pasteurized apple juice, 500 mg/L supplemented melatonin also significantly inhibited the browning, which was also consistent with the antioxidant capacities detected (Figure 6). The effect of 250 mg/L melatonin on inhibiting juice browning was not obvious as in the pasteurized juice. The pasteurization causes Maillard reactions which create more anti-oxidant chemicals and possibly consume less melatonin [51]. Most importantly, a powerful anti-microorganism activity of melatonin was uncovered during juice storage, although melatonin is a molecule frequently reported to reduce the biotic stress and inhibit the growth of microorganisms in plants [75,76,77,78,79,80,81,82,83,84,85], this effect on food storage was not known, especially in the fresh juice with a shelf life of less than 4 hours. Our results showed that melatonin significantly inhibited the total microorganisms, including bacteria, moulds and yeasts during the juice storage during the tested time from 4 to 12 h (Figure 7). According to the national guidelines regarding the number of microorganisms in juice [86,87], fresh apple juice can’t be sold eight hours after juicing at room temperature (25 °C ± 2 °C). However, when melatonin (250 or 500 mg/L) was added, judging from its microorganism number, the apple juice was still safe to be consumed. We observed that melatonin content gradually declined in juice with time. This is consistent with its effect on the juice browning, i.e., melatonin consumption by interaction with oxidants reduces the juice browning. The exact mechanisms of how melatonin exhibits its powerful anti-microorganism effect are currently unknown and they are our future research subject.

In summary, melatonin was detected in the apple juice and may serve as a naturally occurring melatonin supplement in the future. Melatonin not only reduces the apple juice browning by its potent antioxidant capacity, but also inhibits the growth of a wide spectrum of microorganisms including yeasts, moulds and bacteria. These effects of melatonin would preserve the appearance and prolong the shelf life of apple juice. 

## 4. Materials and Methods

### 4.1. Chemicals and Reagents

Melatonin (≥98%) was purchased from Sigma (St. Louis, MO, USA). Folin-Ciocalteau phenol reagent, gallic acid, ABTS, glucose and chloramphenicol were supplied by Sangon Biotech (Shanghai) Co., Ltd. (Shanghai, China). Methanol, ethyl alcohol, sodium carbonate and potassium persulfate were purchased from Beijing Guangda Smirnov Technology Co., Ltd. (Beijing, China). Trolox, tryptone and yeast powder were obtained from Beijing Jingrijindian Science and Technology Ltd. (Beijing, China). Agar was purchased from Beijing AUKS Technology Co., Ltd. (Beijing, China). All reagents were of analytical grade, except the methanol which was of chromatographic grade. 

### 4.2. Growth Conditions

‘Fuji’ apple (*Malus pumila* Mill.) fruits were collected from 9-year-old ‘Fuji’ apple trees which were grafted onto ‘Rehd’ (*Malus robusta* Carr.) stocks and planted at the Jinguoshu Science and Technology Center in Beijing, located at 39°96’ N and 116°22’ W, on October 25th in 2016 and 2017. Apple fruits of the other cultivars (‘Baishaguo’, ‘Orin’, ‘Ralls’, ‘Granny Smith’, ‘Changfu 2’, ‘Ben Davis’, ‘Golden Delicious’, ‘Jiguan, Shanfu 2’, ‘4-23’, ‘Xiaoshuai’, ‘Jonagold’, ‘Stark Jumbo’, ‘Tompkin’s King’, ‘Shinsekai 1’, ‘Jinhong’ and ‘Jincui’) were collected from the 5~8-year-old apple trees planted in the Bakou Apple Orchard in Beijing, on September 16th in 2017. 

### 4.3. Preparation of Apple Peel, Flesh and Juice

The apple fruit was peeled and the skin and flesh was prepared after removing the apple core and slicing; the samples were quick-frozen with liquid nitrogen, and then were kept at −80 °C; (shown in Figure 1a). Apple juice was made by juicing for 30–40 s (220 V, 50 Hz, 240 W) in a juicer (Fissler-7100, Ida-oberstein, Germany), and then it was collected into 10 mL centrifuge tubes followed by quick-freezing with liquid nitrogen. Two apples were used for the preparation of each sample of the peel, flesh and juice for the melatonin detection among the different cultivars. For ‘Fuji’ cultivar, total phenols were also detected. Three duplicate experiments were independently conducted in each cultivar. 

### 4.4. The Melatonin Supplementation and Juice Pasteurization 

Three apples were weighed and used for ‘Fuji’ apple juicing. To detect the melatonin reduction after juicing and storage, 50 and 500 mg/L melatonin were added in the juice in 7:100 (*v*/*w*), respectively. Based on the result of this first melatonin supplementation, in the following experiment to check the juice browning the antioxidant ability of melatonin in juice, before juicing, 250 or 500 mg/L melatonin dissolved into water was added in 7:100 (*v*/*w*) [88]. The process of juicing lasted 30–40 s (220 V, 50 Hz, 240 W) in the juicer (Fissler-7100). After juicing, half of the juice of the control and the two samples supplemented with melatonin were immediately pasteurized at 85–90 °C for 20 min. After the juicing, it took a total of 30 min for the treatment before the juice was frozen for melatonin detection. The other unpasteurized half of the juice was also left at room temperature for about half hour as well. After pasteurization, all the unpasteurized/pasteurized apple juice was frozen immediately. Three duplicate experiments were independently conducted with or without pasteurization. In addition, the final concentrations of melatonin in all samples were analyzed after juicing to compare anti-oxidative capacity of different melatonin levels in apple juice. 

### 4.5. Melatonin and Total Phenols Detection of Apple Fruit 

#### 4.5.1. Melatonin Detection 

A total of 1.5–2.0 g frozen apple peel or flesh, respectively, were ground to a fine powder in liquid nitrogen with a mortar and pestle and suspended in 5 mL methanol and ultrasonicated (at 80 Hz) for 35 min at 45 °C. After centrifugation at 10,000 g at 4 °C for 15 min, the supernatants were collected and dried under nitrogen gas. The extracts were dissolved in 2 mL 5% methanol and transferred to a C18 solid phase extraction (SPE) cartridges (Waters, Milford, MA, USA), which were activated with 2 mL methanol and 2 mL water respectively, for the purification of melatonin. Then the cartridges were washed with 2 mL 5% methanol and melatonin was eluted at a low flow rate with 2 mL 80% methanol, and filtered through 0.2 μm nylon membrane filters (Titan Syringe Filters, Sun Sri, Rockwood, TN, USA). The purified extracts were subjected to HPLC analysis [89,90]. The fruit juice (1 mL) was diluted with 5 mL methanol and then melatonin was detected as the method described above. The melatonin detection of each sample was independently repeated three times.

The recovery rate was determined by adding 100 μL 1 μg/mL standard melatonin (Sigma) solution into the suspension of samples before melatonin extraction using the methods described by Zhao [89]. The melatonin-spiked plant extracts were then analyzed by HPLC.

#### 4.5.2. Total Phenols Detection 

The total phenolic content (TPC) was assessed using the Folin-Ciocalteau phenol reagent method [91,92] with a slight modification. A total of 3.0 g frozen apple peel or 5.0 g frozen fruit flesh, were ground to a fine powder in liquid nitrogen with a mortar and pestle and suspended in 20 mL 80% (*v*/*v*) methanol for 15 min at room temperature, respectively. Then they were diluted with 30 mL 50% (*v*/*v*) methanol and centrifuged for 15 min at 6000 rpm at room temperature. Then 4.8 mL distilled water and 300 μL of Folin-Ciocalteau reagent were added to 300 μL of the extracted sample. After 8 minutes of vortex oscillation at room temperature, 600 μL of sodium carbonate (1 N) were added. The extracts were mixed and kept in the dark at room temperature for 2 h before measuring the absorbance at 725 nm using a UV–visible spectrophotometer (Thermo Scientific Technologies, Madison, WI, USA). A mixture of water and reagents was used as a blank. Gallic acid standard curve (0.02−0.10 mg/mL) was used and the total phenolic content was expressed as milligram gallic acid equivalents per gram fresh weight (mgGAE/gFW) of samples. Each experiment was independently repeated three times. 

### 4.6. Browning Observation and the Browning Index Detection of Apple Juice 

#### 4.6.1. Browning Observation 

The browning of unpasteurized/pasteurized apple juice was observed in 10 mL glass tubes. Photos were taken at hour 0, 2, 4, 8, 12 after juicing using camera (Nikon D7000, Tokyo, Japan). 

#### 4.6.2. Browning Index Detection

The browning index (BI) was measured using the following method [93,94,95]. A total of 10 mL of apple juice was centrifuged for 2 min at 8000 rpm and at room temperature 25 °C ± 2 °C to remove coarse particles, and then 5 mL of absolute ethyl alcohol was added to 5mL of supernatant before the absorbance of the obtained supernatant measured at 420 nm using a UV–visible spectrophotometer (Thermo Scientific Technologies). The browning index of pasteurized and unpasteurized apple juice was both determined at hour 0, hour 2, 4, 8 and 12 after juicing. Each measurement was repeated three times independently.

### 4.7. Detection of Antioxidant Capacity of Apple Juice by the ABTS Assay 

The free radical scavenging activity of the apple juice with different concentrations of melatonin was measured using ABTS reagent, which was prepared by mixing 5 mL 7.4 mM ABTS solution and 5 mL 2.6 mM potassium persulfate solution together to make them react for 16 h at room temperature 25 °C ± 2 °C; in the dark [96]. Then a total of 20 µL apple juice was added to a 1950 µL ABTS reagent to react for 6mins. The absorbance was determined at 734 nm using a UV–visible spectrophotometer (Thermo Scientific Technologies). The free radical scavenging activity was compared to a standard curve of prepared Trolox solutions. Each experiment was independently repeated three times.

### 4.8. The inhibition of Melatonin on Microorganisms 

Total colonies were counted in on plate count agar (PCA) [86,97], following inoculation with 100 μL of apple juice supplemented with 0, 250 and 500 mg/L melatonin or its serial dilutions in physiological saline incubated at 36 °C ± 1 °C for 48 h ± 2 h. Mould and yeast counts were calculated in potato dextrose agar (PDA) [88], following inoculation with 100 μL of apple juice with different concentrations of melatonin or its serial dilutions in sterile water incubated at 28 °C ± 1 °C for 5 days. The results were expressed as colony forming units per milliliter of juice (CFU/mL). Three duplicates were independently conducted.

### 4.9. Statistical Analysis

The data were subjected to ANOVA followed by Fisher’s least significant difference (LSD) or Student’s *t*-test analysis. Statistically significant differences were indicated at *p* < 0.05. Statistical computations were carried out using the SPSS 20.0 software (IBM, Armonk, NY, USA).

## Supplementary Materials

Supplementary Materials are available online.
