# Supplementary material for: Melatonin in Apples and Juice: Inhibition of Browning and Microorganism Growth in Apple Juice
Source: Molecules. 2018 Feb 27;23(3):521. doi: 10.3390/molecules23030521 (PMC6017754; doi:10.3390/molecules23030521)
Supplement: Supplementary file 1 [file molecules-23-00521-s001.pdf]

Table 1. Melatonin levels in the peel of different cultivars

Melatonin levels	Cultivars
Super-high level (70-110ng/g)	'Shinsekai 1', 'Jinhong', 'Jincui'
High level (45-70ng/g)	'Jonagold', 'Stark Jumbo', 'Tompkin's King', 'Fuji'
Medium level (20-45ng/g)	'Jiguan', 'Shanfu 2', '4-23', 'Xiaoshuai'
Low level (0-20ng/g)	'Baishaguo', 'Orin', 'Ralls', 'Granny Smith', 'Changfu 2', 'Ben Davis', 'Golden Delicious'

Table 2. Melatonin levels in the flesh of different cultivars

Melatonin levels	Cultivars
Super-high level (110-150ng/g)	'Shinsekai 1'
High level (45-110ng/g)	'Jinhong'
Medium level (20-45ng/g)	'Jonagold', 'Stark Jumbo', 'Jincui', 'Xiaoshuai', '4-23', 'Tompkin's King', 'Shanfu 2'
Low level (0-20ng/g)	'Fuji', 'Ralls', 'Granny Smith', 'Orin', 'Jiguan', 'Baishaguo', 'Ben Davis', 'Golden Delicious', 'Changfu 2'
